# Mn bioavailability by polarized Caco-2 cells: comparison between Mn gluconate and Mn oxyprolinate

**DOI:** 10.1186/1475-2891-10-77

**Published:** 2011-07-25

**Authors:** Chiara Foglieni, Mariangela Cavarelli, Mariarosaria Piscopiello, Alessandro Fulgenzi, Maria Elena Ferrero

**Affiliations:** 1 Viral Evolution and Transmission Unit, San Raffaele Scientific Institute, Milan, Italy; 2 INSPE, San Raffaele Scientific Institute, Milan, Italy; 3 Department of Human Morphology "Città Studi", Università degli Studi di Milano, Milan, Italy

## Abstract

**Background:**

Micronutrient inadequate intake is responsible of pathological deficiencies and there is a need of assessing the effectiveness of metal supplementation, frequently proposed to rebalance poor diets. Manganese (Mn) is present in many enzymatic intracellular systems crucial for the regulation of cell metabolism, and is contained in commercially available metal supplements.

**Methods:**

We compared the effects of two different commercial Mn forms, gluconate (MnGluc) and oxyprolinate (MnOxP). For this purpose we used the polarized Caco-2 cells cultured on transwell filters, an established in vitro model of intestinal epithelium. Since micronutrient deficiency may accelerate mitochondrial efficiency, the mitochondrial response of these cells, in the presence of MnGluc and MnOxP, by microscopy methods and by ATP luminescence assay was used.

**Results:**

In the presence of both MnOxP and MnGluc a sustained mitochondrial activity was shown by mitoTraker labeling (indicative of mitochondrial respiration), but ATP intracellular content remained comparable to untreated cells only in the presence of MnOxP. In addition MnOxP transiently up-regulated the antioxidant enzyme Mn superoxide dismutase more efficiently than MnGluc. Both metal treatments preserved NADH and βNADPH diaphorase oxidative activity, avoided mitochondrial dysfunction, as assessed by the absence of a sustained phosphoERK activation, and were able to maintain cell viability.

**Conclusions:**

Collectively, our data indicate that MnOxP and MnGluc, and primarily the former, produce a moderate and safe modification of Caco-2 cell metabolism, by activating positive enzymatic mechanisms, thus could contribute to long-term maintenance of cell homeostasis.

## Background

Inadequate dietary intake of micronutrients (i.e. essential minerals, vitamins and other compounds, as mitochondrial metabolites) increases the risk of many degenerative diseases [1]. Micronutrient deficiencies may accelerate chronic metabolic disruption including mitochondrial decay, associated with aging diseases as cancer, heart disease, diabetes and neurodegenerative processes [1]. Specific micronutrient restrictions seem to be related to metabolic alterations affecting specific functions. It is known that moderate to severe Zinc deficiencies influence the immune functions in humans and in animal models [2,3]. More recently, it has been shown that Zinc exerts an important role in the mechanisms of host defense in humans and Zinc supplementation has been successfully used as a therapeutic and preventive agent [4]. In addition, the contribution of microminerals as Iron and Copper to the maintenance of the balance between immunity and health in humans has been demonstrated [5]. Noteworthy, increasing the immunocompetence can decrease the risk of inflammatory disorders, infectious diseases and cancer. However, the overload of micronutrient is toxic but the early effects of these compounds on cell metabolism are still poorly investigated. The use of micronutrient supplementation in adults as part of short-term nutrition therapy is acquiring importance in the metabolic support of patients [6].

Among the essential minerals present in the commercially available nutritional and health supplements there is Manganese (Mn). Mn is involved in enzymatic systems regulating the production of energy, protein metabolism, bone formation, and synthesis of L-dopamine, cholesterol and mucopolysaccharides. Divalent Mn may function as antioxidant, by increasing the scavenging activity of Mn-superoxide dismutase (MnSOD), the principal antioxidant enzyme of mitochondria [7]. Indeed, Mn supplementation may help in the maintenance of cell homeostasis, through the modulation of mitochondrial bioenergetics, thus playing a protective effect against acute inflammation, and likely contributing to pain reduction [8-10]. It has been demonstrated that dietary inorganic nitrates enhance muscle mitochondrial efficiency, increasing the amount of ATP generated [8-10]. Mitochondrial failure and oxidative stress have been postulated as major events in cell aging and death [11]. Hence, the modulation of mitochondrial activity through the assumption of antioxidant minerals from food might help to reduce the risk of chronic diseases of aging [12].

Following oral administration and before absorption, epithelial cells of the small intestine represent the first barrier encountered by Mn and other minerals. Previous study demonstrates a net polarized transport across intestinal epithelial cells for organic but not inorganic Selenium salts, indicative of a different pathway for carbon containing respect to carbon non-containing compounds [13]. More recently effect of bioactive dietary poliphenols on zinc transport across the intestinal Caco-2 cell monolayers has been studied [14]. Conversely, despite the important role exerted by Mn compounds in cell metabolism, the efficacy of Mn supplementation is poorly studied. We hypothesized that Mn supplements might influence the metabolism of intestinal epithelial cells, possibly by increasing their mitochondrial activity. To test our hypothesis, we used an established *in vitro *model of polarized intestinal epithelium which mimics the gut mucosa *in vivo *barrier [13,15,16], e.g. Caco-2 cells cultured in transwell. In this article we focused on two commercially available organic Mn supplements, Mn gluconate (MnGluc), and Mn Oxyprolinate (MnOxP). The metal complexes of Gluconic acid are highly absorbed by small intestine [17,18]. MnOxP, which conjugate Mn to a proline analog, 5-oxyproline, [19] as well as proline-rich peptides, have been proposed to be useful intracellular delivery vectors [20]. Specific objective of our study was to evidence the effects of MnGluc and MnOxP on Caco-2 cell metabolism, in particular on mitochondrial function. Moreover, we have compared the possible different activity of two compounds.

## Methods

### Cells culture and monolayer quality control

Caco-2 cells were cultured on transwell polycarbonate membranes in Dulbecco's modified Eagle's medium supplemented with 10% fetal calf serum and nonessential amino acids to acquire polarization and reach confluence [15]. The electric resistance evaluated the cell continuity; a transepithelial electric resistance ≥330 Ω/cm2 was required to obtain a tight monolayer [21]. In all the experiments the establishment of tight junctions [22] and the development of apical brush border [23] were determined by confocal microscopy in two filters randomly selected. These filters, upon fixation in 2% paraformaldehyde, were labeled with undiluted mouse-anti-human Junctional Adhesion Molecule (JAM, clone BV16, kind gift of Dr. Elisabetta Dejana, IFOM, The FIRC Institute of Molecular Oncology Foundation, Milan) [24] or mouse-anti-dipeptidyl-peptidase type IV (1:100, kind gift of Dr. Ralph Witzgall Institute of Anatomy and Cell Biology I, University of Heidelberg), both overnight at 4°C, revealed by goat-anti-mouse IgG AlexaFluor-594 (1:500, 45 min, Molecular Probes, Eugene, OR). Following metal supplementation studies, randomly chosen specimens were frozen, crio-sectioned, and 10 mm thick transverse sections were stained with Hematoxylin and Eosin to verify the morphology preservation at light microscope (Eclipse 55i, Nikon, Tokyo, Japan).

### Cell labeling for metal supplements study

Filters were divided in two parts. One half was stored for enzymatic assays, the other labeled with mitoTraker CMX-ROS (MT, 100 nM, 45 min, 37°C, Molecular Probes, Eugene, OR) and with acridine orange (AO, 670 nM, 10 min, 37°C, Sigma-Aldrich, St. Louis, Missouri) diluted in warm cell culture medium, to detect mitochondria oxidative activity [25] and cell viability [26], respectively. After rinsing, fresh culture medium containing MnOxP or MnGluc was added into transwell upper chamber.

To obtain a reproducible response with a low standard deviation, a dose-response was performed referring to previous papers on Mn intracellular transport, using 7 μM, 12.5 μM and 25 μM of MnGluc or of MnOxP for 30 min, which contained Mn in quantities well tolerated by Caco-2 cells [13,16].

Subsequently, time-course experiments were performed, treating the polarized Caco-2 cells with 12.5 μM of the two metal compounds for < 5, 15, 30, 60, 120 min, and for 24 h. Experiments were threefold repeated. Filters were fixed in 2% paraformaldehyde, rinsed in PBS and mounted on glass slides for confocal analysis.

Several fixed filters, metal supplemented but unlabeled, were doubly immunostained with Mouse-anti-phospho-ERK (pERK, diluted 1:250, 120 min; sc7383, Santa Cruz Biotechnology, Santa Cruz, CA) and Rabbit-anti-MnSOD (diluted 1:50, 4°C, overnight; Upstate Millipore, Milan, Italy) revealed by Goat-anti-Mouse IgG AlexaFluor594 and Donkey-anti-Rabbit AlexaFluor488 (both diluted 1:500, 45 min, Molecular Probes Invitrogen, Eugene, OR), respectively.

Untreated cells stained with AO/MT or with secondary antibodies only, and unstained monolayers excluded the presence of fluorescent signals either due to metal compounds or to autofluorescence.

### Confocal microscopy

Filters were examined within 24 h from labeling by Leica TCS SP (Leica Microsystems GmbH, Wetzlar Germany) confocal microscope: Z-series were collected from single channels, processed to obtain 2D free projection max (FPM) or cross-sectional images by Leica Confocal Software, then merged by Adobe Photoshop CS. Root Mean Square (RMS) of Fluorescence Intensity was measured in at least three fields/filter at objective x40 in AO/MT specimens.

### ATP assay

ATP content was determined intracellularly and into both apical and basal supernatants from Caco-2 cells submitted to metal supplementation for 5 min, 30 min and 24 h by bioluminescence-based assay (ATP Determination Kit, Molecular Probes, Eugene, OR). Specimens were run in triplicate and the amount of ATP calculated vs. a standard curve for a series of ATP concentrations.

### βNADPH-diaphorase and NADH-diaphorase

Histochemical assays for βNADPH-diaphorase and NADH-diaphorase activities were performed as previously described [27,28] in 4 sets of half-filters, to determine the contribution of nitric oxide synthase-related oxidative activity and MnSOD immunoreactivity. The presence of Blue Tetrazolium salts inside Caco-2 cells was evaluated under light microscope (Eclipse55i, Nikon, Tokyo, Japan).

### Statistical analysis

Statistical comparisons among the RMS data were performed using the JMP4 statistical package (SAS Institute, Cary, NC) and Wilcoxon signed-ranks test. Probability values p < 0.05 were considered significant.

## Results

### Intestinal epithelial cell model: quality control

**Figure 1:**
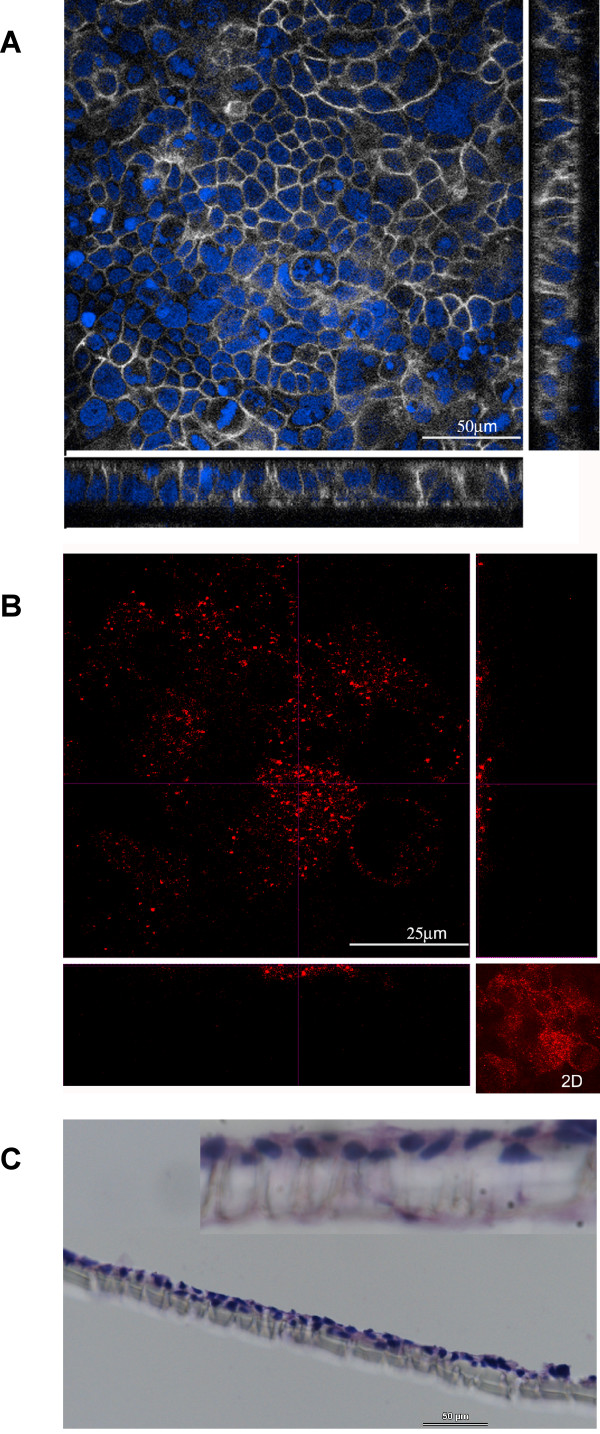
**Microscopy characterization of the *in vitro *intestinal epithelium model**. Cross-sectional images from Caco-2 grown on Transwell filters to form confluent monolayers of differentiated cell are in panels A and B. A junction -rich, organized field is in panel A (JAM white, DAPI blue). Another field with well-developed apical brush border (DPPIV red) is in panel B. Inset in the bottom right corner of panel B displays the 2D free projection max of the same. In panel C Hematoxylin and Eosin stain shows a transversal cryosection from a filter with cells treated with MnOxP for 30 min. The morphology, i.e. columnar cells forming a tight and regular monolayer, appears preserved.

Confluent monolayers of differentiated Caco-2 cells are known as useful model to mimic intestinal epithelium, when cultured on transwell under tightly controlled conditions [15]. Confluency was tested by measuring transepithelial electric resistance, and differentiation by verifying the presence of tight junctions and brush border, stained with anti-JAM (Figure 1A) and anti-dipeptidyl-peptidase type IV (Figure 1B), respectively. Confocal microscopy demonstrated a regular monolayer of correctly polarized cells with well-developed tight junctions along the lateral and apical cell membrane contour, and clear microvilli on the apical surface. Treatment of Caco-2 cells with MnOxP and MnGluc did not alter the monolayer and no sign of apoptosis was observed, as revealed by Hematoxylin and Eosin stain (Figure 1C).

In all specimens, the AO (an indicator of cell viability) brightly labeled the nuclei, without difference in respect to nuclear condensation or chromatin fragmentation. These findings indicated no increase in apoptotic events and a good cell viability [26], thus supporting the morphological findings (not shown). MnOxP and MnGluc were undetectable inside the cells either under visible light or fluorescence (not shown). Moreover, none of the Caco-2 monolayers displayed fluorescence after treatment with these compounds.

### Confocal microscopy of Caco-2 cells submitted to metal supplementation: mitochondrial activity

**Figure 2:**
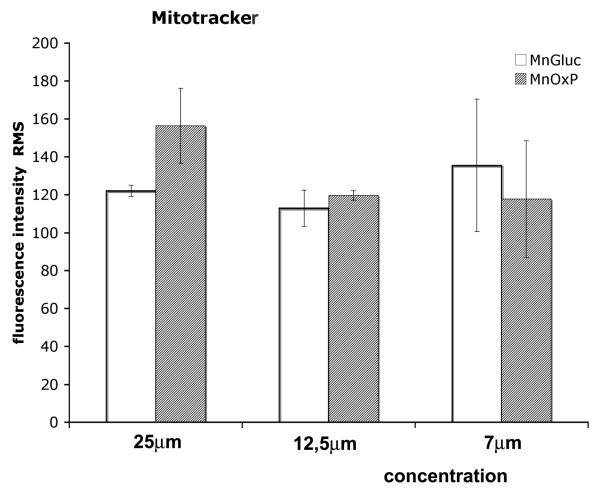
**Confocal microscopy of MT on polarized Caco-2 monolayers**. Root Mean Square fluorescence intensity quantification from dose-response experiment using cells incubated with MnGluc or MnOxP is shown. Bars indicate standard deviation, lowest at the intermediate concentration.

We hypothesized that treatment with metal supplements is able to induce a fast and transient change in MT kinetics. To set up the system, we compared the effects of three different concentrations MnOxP or MnGluc after 30 min incubation. Root mean square (RMS) fluorescence signals showed low standard deviation, indicative of homogeneous intra-sample response, when Mn compounds were used at concentration 12,5 μM (Figure 2): the same was employed in further experiments.

**Figure 3:**
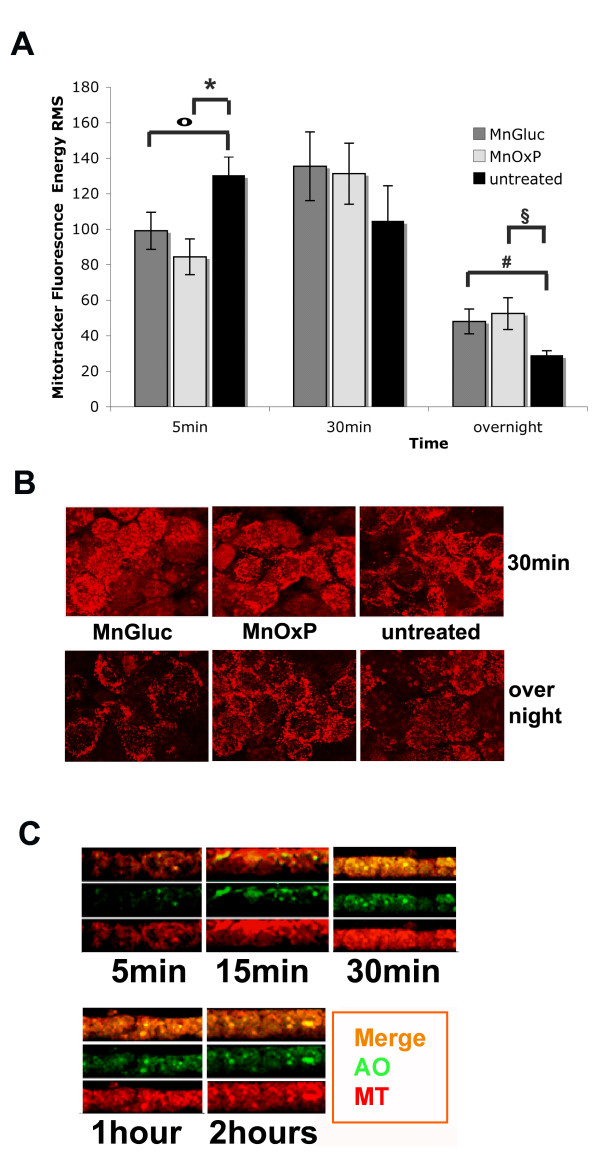
**Confocal microscopy of polarized Caco-2 cells treated with MnGluc or MnOxP- analysis of MT fluorescence**. Quantification and distribution of MT in response to 12.5 μM MnGluc or MnOxP are shown. Root Mean Square fluorescence intensity quantification from a representative long experiment is in panel A. Symbols (o, #, *, §) indicate significant differences (p < 0.05) between Caco-2 cells responses. Panel B shows confocal MT images from the same samples at 30 min and 24 h, in agreement with data in panel A and displaying that the regular mitochondria distribution is preserved in treated cells. Transversal 2D free projection max (panel C) from a brief experiment displays both AO and MT signals in the presence of MnOxP. MT shows a transient redistribution of mitochondria close to the apex of cells at 15 min. AO helps in localize the nuclei respect to MT and demonstrates the cells viability.

Two different series of experiments were performed with MT: the first lasted up to 2 hours (brief) and the others lasted 24 hours (long). Figure 3A displayed the quantitative results obtained with MT. In brief experiments, untreated Caco-2 cells showed elevated RMS for MT fluorescence, indicative of active mitochondria. Results obtained at 5 min and 30 min with and without Mn supplementation were displayed. The reduction of MT signal was significant at 5 min of both MnGluc (p = 0.04) and MnOxP (p = 0.015) treatments respect to untreated cells, possibly related to the compound entry inside the cells. No significant differences were evidenced at 30 min. However, in long experiments, performed by using cells treated either with MnGluc or MnOxP for 24 hours, RMS of MT was slightly higher than in untreated Caco-2 cells (p = 0.010 with both compounds) (Figure 3A). No significant quantitative difference between MnGluc and MnOxP-treated Caco-2 monolayers was found for MT in any time of treatment. Confocal microscopy displayed the qualitative distribution of MT signal at 30 min and overnight, confirming previous observations (Figure 3B). In addition, mitochondria of Caco-2 cells treated with MnOxP overnight resembled to those of untreated 30 min cells, possibly indicating a protective effect of MnOxP. Figure 3C shows the distribution of labeled mitochondria by MnOxP treated Caco-2 cells in brief experiments. At 15 min from the addition of MnOxP to Caco-2 cells MT labeled mitochondria tended to localize close to the apical surface, then (at 30 min, 1 hour and 2 hours) redistributed into the cytoplasm. On the contrary, in MnGluc-treated cells, no change in mitochondrial distribution was observed (not shown).

### ATP content

**Figure 4:**
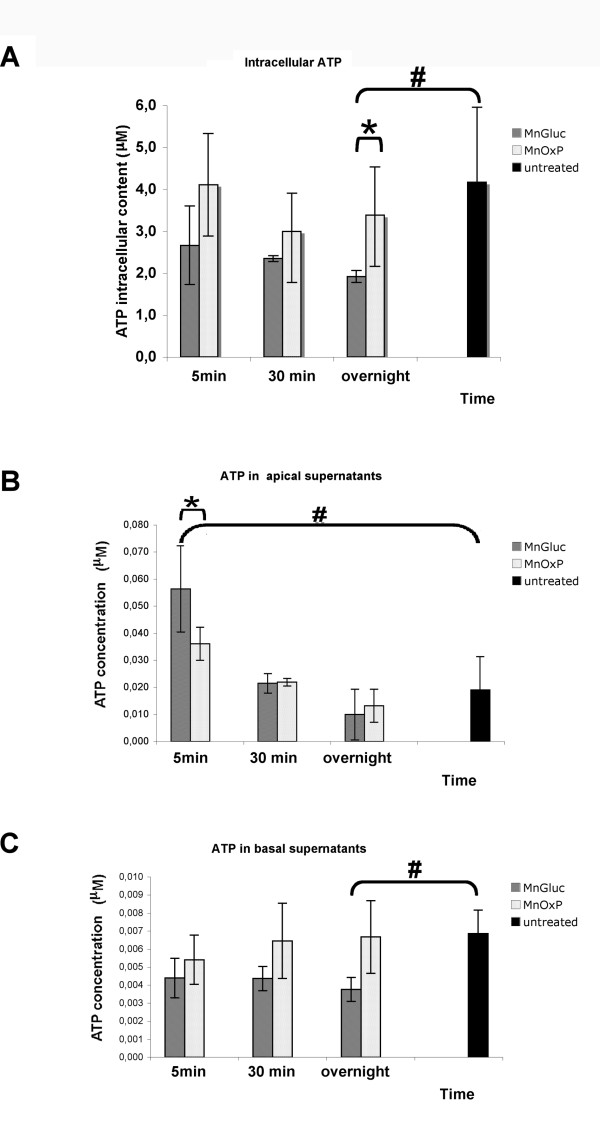
**ATP measurement in Caco-2 cell treated with MnGluc or MnOxP**. Panel A shows the quantification of ATP in cell homogenates. No difference between MnOxP -treated compared to untreated cells was measured. Treatment with MnGluc diminished the intracellular content of ATP at 24 h, respect to both MnOxP-treated and untreated Caco-2 cells. ATP determination in apical supernatants of Caco-2 cells in transwell system cell displays that ATP release during the early times (5 min) of MnGluc supplementation is significantly higher in comparison with both MnOxP-supplemented and untreated cells (panel B). In the basal supernatants the release of ATP is in nanomolar range, which renders the statistical difference between MnGluc-treated and untreated cells showed in the histogram not physiologically relevant (panel C). Symbols: * indicates MnGluc differences (p < 0.05) respect to MnOxP, **# **respect to untreated cells.

The intracellular content of ATP (Figure 4A) was significantly higher in control and MnOxP with respect to MnGluc-treated Caco-2 cells (p < 0.05 for both) following overnight treatment. In polarized cell monolayers the ATP release is directed toward the apical extracellular space, thus ATP released by Caco-2 into the apical supernatant (i.e. culture medium in the transwell upper chamber) was one hundred fold less than intracellular ATP (Figure 4B). During the first minutes of metal supplementation, the monolayers treated with MnGluc released apically around 2 fold more ATP than cells incubated with MnOxP (p < 0.05). Successively (after 30 min and overnight treatments) ATP amount of all treated cells decreased to that of untreated cells (Figure 4B). Low levels of ATP were detected in basal supernatants of treated cells without significant differences with untreated cells, except for slight (< 0.05) ATP decrease in overnight-treated MnGluc cells (Figure 4C), indicating that no abnormal ATP secretion is occurring in our system.

### Confocal microscopy of Caco-2 cells submitted to metal supplementation: MnSOD/pERK

**Figure 5:**
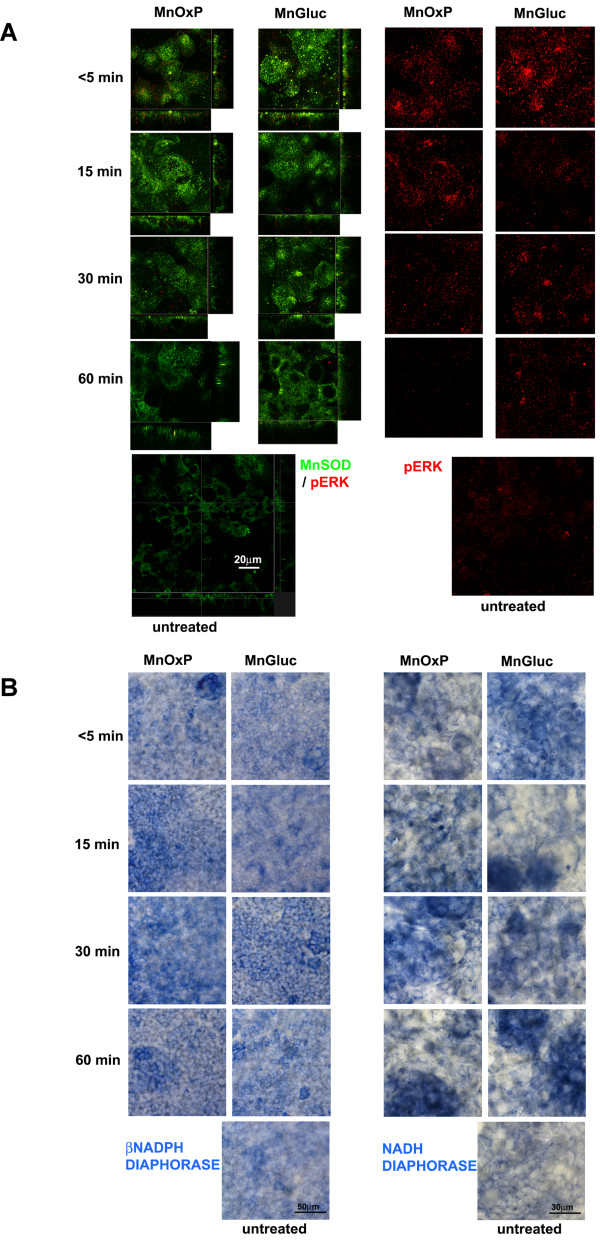
**Confocal microscopyof* pERK, MnSOD *and diaphorase histochemistryon *Caco-2 *treated with* MnGluc *or *MnOxP***. Single plane cross-sectional images of a z-series stack from Caco-2 cells doubly labeled for MnSOD (green) and pERK (red) are in panel A left. Cross sections show x-y (squared), x-z and y-z (both rectangular) projections of the same field. Bright granular fluorescence for MnSOD with both metal supplements is shown, but only faint granules of pERK are visible, indicative of mitochondria metabolically active and of healthy cells, respectively. In panel A right the 2D free projection max obtained from all z-planes of the stack are shown, to evidence the contribution of whole cells to pERK signal and to underline the scarce total expression of pERK. βNADPH- and NADH diaphorase assays (representative experiment in panel B) demonstrates by the presence of stable blue formazan precipitate a prevalent activation of NADH- respect to βNADPH-related enzymes upon incubation with MnGluc or MnOxP, a slightly different time-course with the two compounds and a moderate but non-homogenous activation of NADH diaphorase in treated compared to untreated cells.

MnSOD was brightly expressed on the apical region of untreated Caco-2, whereas pERK signal was faint. Consequently, Figure 5A showed single cross-sectional plans for double labeling MnSOD and pERK, and 2D FPM images for pERK alone, with the aim to enhance information. In the presence either of MnOxP or MnGluc, few pERK granules were found into cytoplasm, mostly at 5 min, and closely to MnSOD (Figure 5A). In treated cells, pERK fluorescence decreased to a barely detectable signal in 60 min of treatment (Figure 5A, right panels). In the presence of both Mn compounds the signal for MnSOD more homogenously localized in the apical part of the cell cytoplasm respect to untreated cells as demonstrated by x-z and y-z transversal projections (Figure 5A, right panels). Caco-2 monolayers incubated with MnGluc displayed a signal for MnSOD comparable to that of untreated cells. In Caco-2 incubated with MnOxP, MnSOD signal moderately increased at 15 min, then decreased with time.

### βNADPH-diaphorase and NADH-diaphorase

In mitochondrial diaphorase histochemistry, the punctuate blue formazan stain differed for intensity depending on the coenzyme used. The inhomogeneous signal (i.e. its presence in all cells with irregular distribution and intensity) hindered the reliability of a densitometry, limiting to qualitative observation. βNADPH-diaphorase (Figure 5B, left panels), indicative of nitric oxide synthase activity, was weak; a modest increase was observed at 15 and 30 min after MnOxP and at 30 min after MnGluc, in comparison with untreated cells. NADH-diaphorase (Figure 5B, right panels), i.e. MnSOD reactivity, was higher in all treated monolayer than in untreated, frequently showing cluster-restricted greatest activity.

## Discussion

Some minerals and vitamins have been reported as micronutrients whose deficiencies might cause DNA damage and accelerate mitochondrial decay associated to degenerative diseases of aging [29] and cancer [1]. In humans, the mineral absorption could be improved by complexation with organic compounds such as gluconate, as reported for Zinc [30]. However, controversial results accounted for the bioavailability of these salts, indicating a different behavior, which depends on target cells or tissue and on pathological conditions. In anemic cancer patients the intravenous supplementation with Fe-gluconate increased the levels of blood hemoglobin more than oral Iron therapy [31]. However, in healthy female volunteers, orally administrated Zn-bis-glycinate exhibited an higher Zinc bioavailability than Zn-gluconate [32]. Moreover, in the rat liver Zn-enriched and Cu-enriched yeasts were more bioavailable (i.e. absorbed and found detected in a greater concentration) than Zn-gluconate and Cu-gluconate [33]. Therefore, among ten different organic and inorganic Magnesium salts orally administered in Mg-depleted rats, Mg-gluconate exhibited the highest Magnesium bioavailability at intestinal level [34], slightly superior to Mg-pidolate and Mg-citrate.

In the present work we study Mn, another essential mineral that is a required component of a number of mitochondrial enzymes including MnSOD, and whose inadequate levels increase mitochondrial oxidants and subsequent mitochondrial decay [7].

The following points resume the results obtained by treating polarized Caco-2 cells with two different organic Mn salts. 1) MnGluc and MnOxP does not affect cell viability, as demonstrated by unaltered morphology and by the transient raise of pERK expression. 2) In brief experiments, in the presence of MnOxP and MnGluc, the mitochondrial activity is significantly lower with respect to untreated cells at 5 min only. In long experiments (24 h) both metal supplements moderately improve the mitochondrial oxidative activity with respect to untreated Caco-2 cells. 3) In comparison to Caco-2 cells treated with MnOxP and untreated cells, MnGluc-treated cells apically release a higher amount of ATP at 5 min, and display a lower ATP intracellular content at 24 h. 4) In treated cells MnSOD expression is variable but sustained at all time points. 5) Both βNADPH and NADH diaphorase activities are maintained by Caco-2 cells upon metal supplementation and seem more sustained than in untreated cells at few time points.

Taken together, in this *in vitro *model of intestinal epithelium, our findings demonstrate that MnGluc and MnOxP at the concentration of 12.5 μM are able to elicit a rapid metabolic response, which relates to mitochondrial activation. In metal supplemented respect to untreated Caco-2 cells, the mitochondrial oxidative activity is not significantly impaired, but slightly increases after 24 h of treatment, as indicated by MT fluorescence intensity. The limited changes in mitochondrial oxidation, i.e. tendency of MT fluorescence to decrease during brief treatment, appear counter-balanced by MnSOD upregulation in both MnOxP- and MnGluc-treated cells. MnSOD, an antioxidant defense mitochondrial metalloenzyme, is considered a good parameter to monitorate Mn exposure and protection in humans [35]. The increase in MnSOD could scavenge superoxide anion, protecting the cells from oxidative damage and apoptosis, and ultimately self-regulating the oxidative stress [7]. Of note, the mitochondrial activity appears higher in the presence of both MnOxP and MnGluc than in untreated cells, as shown by MT and by MnSOD.

In so far the MnSOD localization has been associated to NADH-diaphorase reactivity on gut tissue [28]. The untreated polarized Caco-2 confluent monolayers do not display elevate histochemical signal for NADH- nor βNADPH-diaphorase, as previously reported for DT-diaphorase [36]. Indeed, in MnOxP- and MnGluc-treated cells NADH-diaphorase is highest and increases with time, whereas βNADPH-diaphorase weakly and transiently increases, suggesting a modest activation of nitric oxide synthase [27]. Although we cannot exclude a contribute of other enzymes of the diaphorase family to enzymatic activity observed in Caco-2 cells, the enhancement following Mn supplementation supports the activation of a reductase-based metabolism protective against oxygen radical production.

The adverse effects of Mn supplementation depend on the cell type (picomoles are toxic for astrocytes [37], whereas micromoles are safe for enterocytes [13]) and on the administration way [38]. In our model, a sustained pERK activation, which is associated to abnormal mitochondria [39] and to superoxide anion production [40], is absent. This is demonstrated by the few more pERK granules transiently appearing inside Caco-2 cytoplasm during the first 15 min of incubation either with MnOxP or MnGluc, which revert within 60 min to the level of untreated monolayers. A transient polarization of mitochondria in MnOxP-treated monolayers accompanied the increase in pERK supportive of a mitochondria response. Indeed, long experiments demonstrated a general preservation of mitochondrial distribution in Mn-supplemented Caco-2 cells with respect to untreated cells. This observation excludes a commitment to cell death via mitochondrial dysfunction, is in agreement with morphological data, and supports the hypothesis that metal supplements may not affect Caco-2 cells viability, i.e. in these conditions MnGluc and MnOxP are not toxic for enterocytes. However, our findings are to be considered preliminary respect to the effects of MnOxP and MnGluc on gut cells in vivo. Although the general principles of nutrient risk assessment have been established, the value of controlled human studies on nutrient metabolism needs to be improved [41]. In the nutritional field, despite the common use of supplements, the dietary quantity is crucial; a safe and adequate daily dietary intake estimated 1.8-2.3 mg Mn/day for adults [42]. High levels of Mn are associated with neurodegenerative phenomena [38], whereas low doses were claimed as beneficial in prevention and treatment of osteoporosis in women [43].

Interestingly MnOxP does not deplete ATP content in Caco-2 cell, whereas MnGluc decreases moderately ATP levels in all the considered compartments. Together with the moderate reduction of mitochondria oxidative activity detected by MT, these findings suggest a decreased production of ATP by still viable Caco-2 in the presence of MnGluc, but an increased production in the presence of MnOxP, less affecting cell healthiness. These results support the hypothesis of a possible better bioavailability *in vivo *for MnOxP than for MnGluc.

## Conclusions

In summary, the treatment of polarized Caco-2 monolayers with MnOxP or MnGluc produces a moderate and safe modification of cell metabolism, with the activation of positive enzymatic mechanisms, for a long-term maintenance of cell homeostasis. This indicates a good metabolic response to both supplements intake in our in vitro model. However, between the two compounds, MnOxP seems to act more rapidly and dissipates less intracellular ATP. In conclusion, Mn supplements and in particular MnOxP, could represent a promising potential target to address pharmacological studies aimed at prevention and treatment of degenerative mitochondrial dysfunction and age-related diseases. Further kinetics and *in vivo *investigations, carefully designed to exclude harm, are required to confirm dietary bioavailability of these compounds.

## Competing interests

The authors declare that they have no competing interests.

## Authors' contributions

CF and MEF were involved in the design of the study and written the manuscript. MC, MP and AF performed laboratory analyses and statistics. All authors read and approved the final manuscript.
